# Efficacy and safety of sivelestat in sepsis-induced acute respiratory distress syndrome: an updated meta-analysis based on disease severity and treatment strategy subgroups

**DOI:** 10.3389/fmed.2026.1986111

**Published:** 2026-10-27

**Authors:** Abduwupuer Haibier, Jiawei Ma, Yuanyuan Wang, Tong Wu

**Affiliations:** 1 Xinjiang Medical University, Urumqi, China; 2 Department of Critical Care Medicine, Wuxi No. 2 People’s Hospital, Jiangnan University Medical Center, Wuxi, China; 3 Department of Critical Care Medicine, Suzhou Research Center of Medical School, Suzhou Hospital, Affiliated Hospital of Medical School, Nanjing University, Suzhou, China

**Keywords:** acute respiratory distress syndrome, meta-analysis, neutrophil elastase, precision medicine, sepsis, sivelestat, subgroup analysis

## Abstract

**Objective:**

Sivelestat sodium hydrate is a selective neutrophil elastase inhibitor that attenuates neutrophil-mediated alveolar–capillary injury and is used in some East Asian countries for acute lung injury/ARDS. We evaluated the efficacy and safety of sivelestat in sepsis-induced acute respiratory distress syndrome (ARDS) and explored whether disease severity, baseline mortality risk, and treatment background modify the mortality effect.

**Methods:**

We searched PubMed/MEDLINE, Embase, Cochrane CENTRAL, Web of Science, CNKI, Wanfang, and ClinicalTrials.gov from database inception to August 27, 2026. Eligible studies were adult studies of sepsis-induced ARDS comparing a sivelestat-containing regimen with an otherwise comparable regimen without sivelestat. COVID-19-only cohorts, non-septic ARDS, mixed-etiology studies without a separable sepsis subgroup, and comparisons in which both groups received sivelestat were excluded. The primary outcome was all-cause mortality; secondary outcomes included P/F ratio, duration of mechanical ventilation, ICU length of stay, organ-function scores, inflammatory markers, and adverse events. Mortality was pooled as odds ratios (ORs) using a random-effects model with restricted maximum likelihood; Hartung–Knapp intervals were used for the primary and study-design analyses. This review was prospectively registered in PROSPERO.

**Results:**

Fourteen studies were eligible for qualitative synthesis, and 10 studies (943 patients) provided extractable 2 × 2 mortality data. Deaths occurred in 73/431 patients receiving sivelestat and 125/512 controls. Sivelestat was associated with lower mortality (OR=0.55, 95% CI 0.36–0.84; I^2^ = 0%). The signal persisted in randomized trials (OR=0.37, 95% CI 0.16–0.86; I^2^ = 0%). Study-level point estimates appeared more favorable in cohorts with baseline P/F < 100 mmHg and control mortality >30%; however, interaction tests were not statistically significant and severity analyses were ecological. Leave-one-out analyses were stable. Egger testing did not detect significant small-study asymmetry (*P* = 0.197), although power was limited. Baseline-stratified treatment effects by IL-6, PCT, CRP, or neutrophil elastase were unavailable for quantitative effect-modification analyses.

**Conclusions:**

In an updated and etiologically strict evidence synthesis, sivelestat was associated with lower mortality in sepsis-induced ARDS, including in randomized trials. Severe hypoxemia and high baseline risk remain plausible enrichment factors, but current aggregate evidence is insufficient to support a validated phenotype-guided prescribing rule. Co-intervention reporting was sparse, and residual confounding by prone positioning, sedation, neuromuscular blockade, and steroid background cannot be excluded.

**Systematic Review Registration:**

PROSPERO CRD420261541158.

## 1 Introduction

Sepsis is life-threatening organ dysfunction caused by a dysregulated host response to infection (1). Acute respiratory distress syndrome (ARDS) is characterized by acute hypoxemic respiratory failure, bilateral pulmonary infiltrates, and noncardiogenic pulmonary edema; the Berlin definition further classifies disease as mild, moderate, or severe according to the PaO_2_/FiO_2_ ratio (2). Sepsis is an important precipitant of ARDS. When the two conditions coexist, inflammatory cascades, endothelial and epithelial injury, coagulation abnormalities, and neutrophil activation amplify one another, leading to prolonged mechanical ventilation and a high risk of death.

In recent years, ARDS research has increasingly shifted from viewing ARDS as a single syndrome toward phenotype- and subphenotype-based stratification. An international Delphi expert panel has suggested that future ARDS trials should place greater emphasis on stratification and enrichment based on biological and clinical characteristics (3). Neutrophil elastase (NE) can degrade the extracellular matrix and aggravate injury to the alveolar-capillary barrier, making it a potential therapeutic target in hyperinflammatory lung injury (4). Sivelestat is a selective NE inhibitor that has accumulated a substantial clinical evidence base in critically ill patients in East Asia.

The restriction to sepsis-induced ARDS was prespecified for both clinical and biological reasons. Sepsis is a leading precipitant of ARDS in large international cohorts, and sepsis-associated ARDS has been consistently reported to have a higher mortality than ARDS from many non-septic causes (5, 6). COVID-19-associated ARDS has distinct immunopathological, thrombotic, and treatment features, including routine corticosteroid and antiviral use, whereas trauma-, pancreatitis-, and surgery-associated ARDS differ in initiating mechanisms and co-interventions. Pooling these heterogeneous etiologies can dilute or distort the treatment effect of an anti-neutrophil-elastase therapy and limits applicability to the specific clinical question of sepsis-induced ARDS. A strict etiologic definition is therefore necessary to estimate the treatment effect in the population in which neutrophil-elastase-driven hyperinflammation is most plausible.

Two recent syntheses are particularly relevant. Zheng et al. (2025) pooled 17 studies and 5,062 patients and reported lower mortality, improved oxygenation, and shorter ventilation/ICU stay with sivelestat, but their population included COVID-19-related, non-septic, and mixed-etiology ARDS, and treatment backgrounds varied substantially (7). Charoensappakit et al. (2026) restricted the analysis to randomized controlled trials but still included acute lung injury/ARDS of mixed etiology (8). Neither review was designed to answer whether sivelestat improves mortality specifically in sepsis-induced ARDS, and neither comprehensively captured Chinese-language studies or studies published after May 2025. An updated, etiologically strict synthesis is therefore needed to reduce clinical heterogeneity and to test whether severity, baseline risk, and background therapy modify the mortality effect.

Accordingly, the present study adopted a strategy of strict etiologic definition, evidence updating, and subgroup exploration. The primary analysis was restricted to sepsis-induced ARDS, with supplementation by Chinese-language studies missed previously and studies published after May 2025. Particular attention was paid to whether study design, severity of hypoxemia, baseline mortality risk, and concomitant treatment background modified the mortality effect. For inflammatory biomarker subgroups, quantitative pooling was undertaken only when the original studies reported treatment effects stratified by baseline phenotype, thereby avoiding the erroneous interpretation of post-treatment biomarker reductions as evidence that a baseline phenotype predicts treatment response.

## 2 Methods

### 2.1 Study design and reporting standards

This study was organized and reported in accordance with the PRISMA 2020 statement (9). This review was prospectively registered in PROSPERO (CRD420261541158). The protocol, including the PICOS framework, eligibility criteria, primary and secondary outcomes, prespecified subgroups, and statistical analysis plan, was specified before literature screening.

### 2.2 Search strategy and time frame

The databases searched included PubMed/MEDLINE, Embase, Cochrane CENTRAL, Web of Science, CNKI, Wanfang, and ClinicalTrials.gov. We searched from database inception to August 27, 2026. A start date of 2015 was not used because it would have excluded earlier studies by Endo, Hayakawa, Tsuboko, and Miyoshi. For studies conducted before the Berlin definition, inclusion was allowed when the original ALI/ARDS diagnostic criteria, hypoxemia criteria, and etiologic information could be reasonably mapped to the modern concept of ARDS; differences arising from the era of ARDS definition were then addressed in sensitivity analyses and the Discussion.

The formal search combined controlled vocabulary and free-text terms and initially use a high-sensitivity strategy without an RCT/cohort study-design filter; study design should then be identified through title/abstract and full-text screening.

### 2.3 PICOS framework and eligibility criteria

Exclusion criteria were COVID-19-only cohorts; non-septic ARDS caused by trauma, pancreatitis, cardiothoracic surgery, or other etiologies; mixed-etiology studies in which the sepsis subgroup could not be separated; comparisons in which both groups received sivelestat; duplicate publications; and studies without usable outcome data. Eligible publication languages were Chinese and English. See Table 1 for details.

**Table 1:** PICOS framework and eligibility criteria.

Dimension	Operational definition used in this study
P (Population)	Adults (>=18 years) with sepsis-induced ARDS; Berlin definition or AECC/ALI-ARDS criteria that can be reasonably mapped to the modern ARDS concept; sepsis must be confirmable as the principal precipitant of ARDS.
I (Intervention)	Sivelestat sodium, with no restriction on dose or duration. Combination with ulinastatin, corticosteroids, specific ventilation strategies, or other therapies was allowed, provided that the control group had the same treatment background and the only reasonable between-group difference was attributable to sivelestat.
C (Comparison)	Placebo, standard care, or the same background combination therapy without sivelestat. Studies in which both groups received sivelestat and differed only by the addition of another drug were excluded from the primary efficacy meta-analysis.
O (Outcomes)	Primary: all-cause mortality (preferentially the longest comparable in-hospital/28-day/reported time point). Secondary: P/F ratio, duration of mechanical ventilation, ICU length of stay, organ-function scores, inflammatory markers, and adverse events.
S (Study design)	RCTs and observational studies with a parallel control group. Case reports, case series, animal studies, reviews, and studies without extractable comparative effects were excluded.

### 2.4 Outcomes and data extraction

Two investigators independently extracted the following information: author, year, country/center, study design, sample size, ARDS definition, source of infection, age, P/F ratio, APACHE II score, SOFA score, control-group mortality risk, sivelestat dose and treatment duration, concomitant interventions, mortality outcomes, oxygenation, duration of mechanical ventilation and ICU stay, biomarkers, and adverse events. In addition, we specifically extracted co-interventions that could confound oxygenation or mortality, including protocolized steroid/anti-inflammatory therapy, prone positioning, sedation, neuromuscular blockade, and the timing of sivelestat initiation relative to proning. When these variables were not reported, they were recorded as “not reported” (NR) and were not imputed.

### 2.5 Risk of bias and certainty of evidence

We planned to assess RCTs using the RoB 2 tool (10), observational studies using ROBINS-I (11), and the certainty of evidence using the GRADE framework across risk of bias, inconsistency, indirectness, imprecision, and publication bias (12). However, because the available reports for some Chinese-language studies lacked sufficient information on allocation concealment, blinding, or completeness of outcome reporting, and because such assessments would require independent full-text verification by two reviewers, we did not present a formal risk-of-bias summary figure or GRADE certainty ratings, thereby avoiding a false impression of precision.

### 2.6 Statistical analysis

Binary mortality outcomes were expressed as odds ratios (ORs) with 95% confidence intervals (CIs). Given clinical heterogeneity in study era, control treatment background, and design, a random-effects model was prespecified for the primary analysis. Between-study variance (tau^2^) was estimated using restricted maximum likelihood (REML); Hartung-Knapp intervals were used for the primary analysis and the study-design subgroup analysis. Heterogeneity was reported using Cochran Q, *I^2^*, and tau^2^.

Prespecified subgroups were: (1) RCTs versus observational studies; (2) study-level baseline *P/F* ratio <100, 100–199, and 200–299 mmHg; (3) control-group mortality >30% versus ≤30%; and (4) background therapy with ulinastatin in both groups versus no such background therapy. Subgroup differences were evaluated by interaction tests. The P/*F* subgroup analysis used study-level mean values and was therefore ecological; it does not represent a patient-level interaction.

Because prone positioning, sedation, neuromuscular blockade, and steroid/anti-inflammatory background therapy were inconsistently reported across included studies, no formal subgroup analysis or meta-regression was performed for these co-interventions. These variables were tabulated descriptively to allow readers to judge the extent of potential confounding and were addressed explicitly in the Limitations.

Sensitivity analysis was performed using a leave-one-out approach. When the number of included studies reached 10, a funnel plot and *Egger* regression were used; because k = 10 is at the lower boundary of conventional applicability, conclusions regarding publication bias were interpreted cautiously. Quantitative pooling of inflammatory phenotypes, infection source, or pathogen subgroups was undertaken only when at least two studies reported comparable baseline-stratified treatment effects. When studies reported only post-treatment mean values of IL-6, PCT, CRP, or NE, these were treated as pharmacodynamic outcomes and were not used for predictive-biomarker meta-analysis.

## 3 Results

### 3.1 Evidence update and study inclusion

Using the 2025 systematic review as the evidence base, studies were first re-screened according to the strict etiologic criteria, followed by supplementary searches for Chinese-language studies and studies published after May 2025. At the study level, 14 studies met the criteria of sepsis-induced ARDS with a comparable control group not receiving sivelestat and were included in the qualitative synthesis. Of these, 10 studies provided verifiable exact mortality event counts and were included in the quantitative mortality meta-analysis, comprising 943 patients. The remaining four studies contributed to the qualitative review of oxygenation, inflammation, or other outcomes, but publicly available information was insufficient to construct reliable 2 × 2 mortality tables. The detailed PRISMA 2020 flow diagram of study identification, screening, and inclusion is presented in Figure 1.

**Figure 1:**
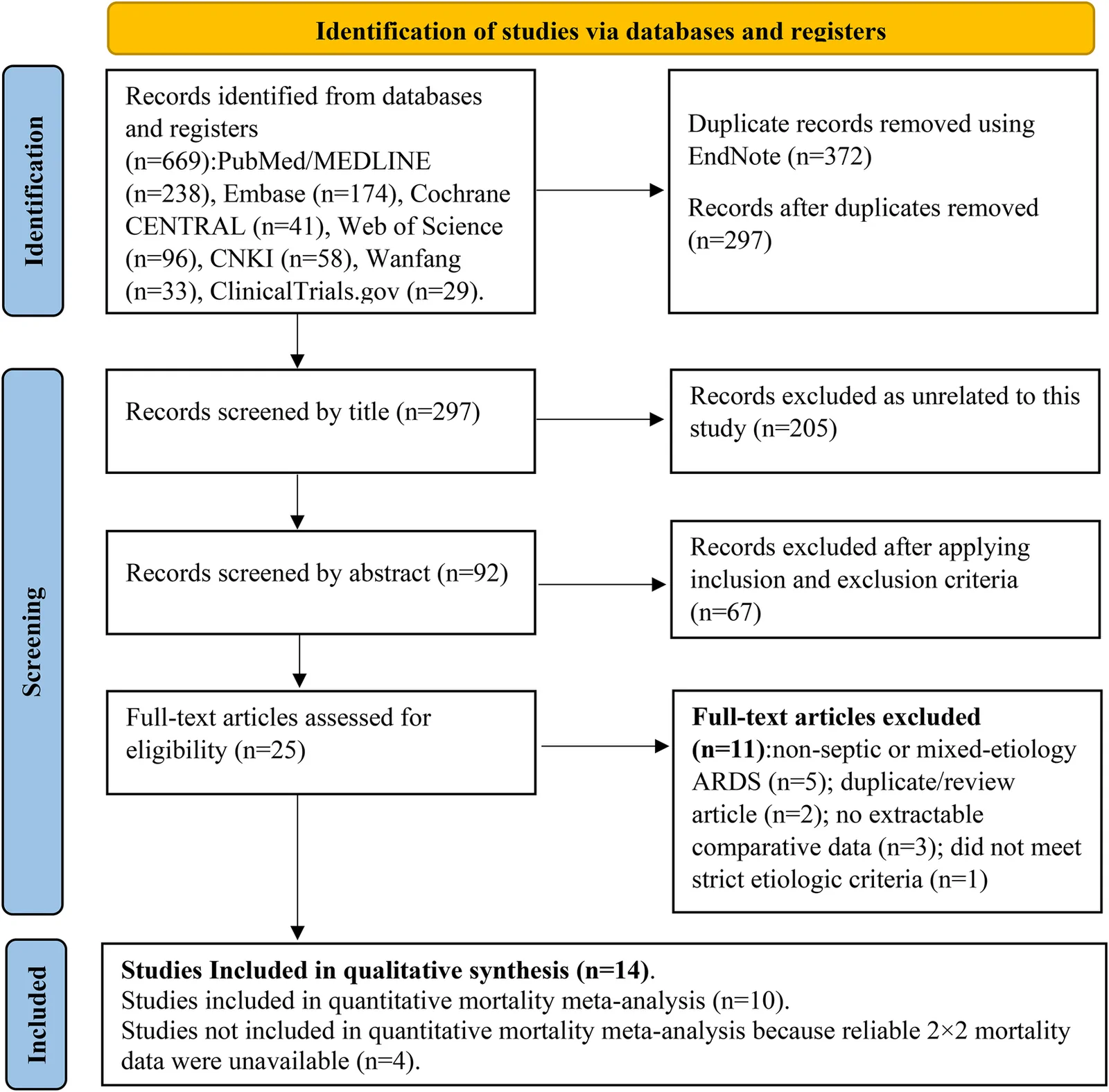
Study selection process for the updated systematic review and meta-analysis of sivelestat in sepsis-induced ARDS.

### 3.2 Characteristics of included studies

Table 2 summarizes the design, sample size, baseline P/F ratio, treatment duration, concomitant background treatment, and mortality events of the included studies. Table 3 details reported steroid/anti-inflammatory background and co-interventions, including prone positioning, sedation/neuromuscular blockade, and the timing of sivelestat initiation relative to proning. Among the 14 studies included in the qualitative synthesis, 10 provided verifiable mortality event counts and entered the quantitative mortality meta-analysis; the remaining four contributed to qualitative synthesis of oxygenation, inflammation, or other outcomes. Co-intervention reporting was sparse, and no quantitative co-intervention-adjusted analysis was performed. All 14 studies in the qualitative synthesis were conducted in East Asia: four in Japan and ten in China. Among the 10 studies included in the quantitative mortality meta-analysis, four were from Japan and six were from China. No eligible studies from Europe, North America, South America, Africa, or other Asian regions were identified.

**Table 2:** Characteristics of included studies.

Study	Design	Sivelestat/control, n	Baseline P/F^a^	Duration	Concomitant treatment background	Mortality events
Endo 2006 (13)	RCT	13/13	NR	14 d	Standard care	1/13 vs 2/13
Hayakawa 2010 (14)	Observational	34/133	121.0	14 d	Standard care	7/34 vs 31/133
Tsuboko 2012 (15)	Observational	34/15	174.4	14 d	Standard postoperative care for abdominal sepsis	2/34 vs 2/15
Miyoshi 2013 (16)	Observational	70/40	154.3	14 d	Standard care	24/70 vs 21/40
Gao 2021 (17)	Observational	60/80	136.8	14 d	Standard care	3/60 vs 5/80
Zhou 2022 (18)	Randomized, 3 arms	40/40^b^	NR	NR	Standard care; separate ulinastatin arm	14-day mortality reported; events NR
Shan 2023 (19)	Randomized	44/44	NR	NR	Standard care	Mortality reported; events NR
Qi 2023 (20)	Observational	70/71	217.2	NR	Standard care	12/70 vs 10/71
Lv 2024 (21)	RCT	35/35	NR	NR	Standard care	Mortality events NR
Xu 2024 (22)	RCT	42/42	77.5	7 d	Ulinastatin in both groups	15/42 vs 22/42
Lin 2024 (23)	Random-number table	30/30	NR	7 d	Standard care	14-day mortality reported; events NR
Wu 2025 (24)	RCT	34/36	148.9	5–14 d	Placebo + standard care	3/34 vs 10/36
Li 2025 (25)	Observational	23/31	92.3	7 d	Methylprednisolone + APRV in both groups	5/23 vs 15/31
Liu 2026 (26)	RCT	51/51	NR	7 d	Ulinastatin in both groups	1/51 vs 7/51

a P/F represents the study-level baseline mean or approximate pooled mean when available and was used only for exploratory severity stratification. NR, insufficient publicly verifiable information.

b Zhou 2022 was a three-arm study; the sivelestat arm and standard-care arm are shown here, with a separate ulinastatin arm. All 14 studies in the table were included in the qualitative synthesis; 10 studies with exact 2 × 2 mortality data were included in the primary meta-analysis.

**Table 3:** Reported steroid/anti-inflammatory background and co-interventions in included studies.

Study	Steroid/anti-inflammatory background	Prone positioning	Sedation/NMBA	Sivelestat timing relative to proning	Notes
Endo 2006 (13)	Standard care; steroid NR	NR	NR	NR	No co-intervention matching reported
Hayakawa 2010 (14)	Standard care; steroid NR	NR	NR	NR	No co-intervention matching reported
Tsuboko 2012 (15)	Standard postoperative care for abdominal sepsis; steroid NR	NR	NR	NR	No co-intervention matching reported
Miyoshi 2013 (16)	Standard care; steroid NR	NR	NR	NR	No co-intervention matching reported
Gao 2021 (17)	Standard care; steroid NR	NR	NR	NR	No co-intervention matching reported
Zhou 2022 (18)	Standard care; separate ulinastatin arm; steroid NR	NR	NR	NR	Three-arm design; mortality events not extractable
Shan 2023 (19)	Standard care; steroid NR	NR	NR	NR	Mortality events not extractable
Qi 2023 (20)	Standard care; steroid NR	NR	NR	NR	No co-intervention matching reported
Lv 2024 (21)	Standard care; steroid NR	NR	NR	NR	Focused on septic cardiomyopathy
Xu 2024 (22)	Ulinastatin in both arms; steroid NR	NR	NR	NR	Ulinastatin background-matched
Lin 2024 (23)	Standard care; steroid NR	NR	NR	NR	Qualitative synthesis
Wu 2025 (24)	Standard care; steroid NR	NR	NR	NR	Multicenter double-blind RCT
Li 2025 (25)	Methylprednisolone + APRV in both arms	NR	NR	NR	Protocolized background in both arms; limits generalizability
Liu 2026 (26)	Ulinastatin in both arms; steroid NR	NR	NR	NR	Ulinastatin background-matched

NR, not reported; NMBA, neuromuscular blocking agent; APRV, airway pressure release ventilation; RCT, randomized controlled trial.

### 3.3 Primary outcome: all-cause mortality

Ten studies included 943 patients, of whom 431 received sivelestat and 73 died (16.9%), while 512 were controls and 125 died (24.4%). Random-effects pooling showed that sivelestat was associated with a lower risk of death (OR=0.55, 95% CI 0.36–0.84; *I^2^* = 0%, Q = 8.60, *P* for heterogeneity = 0.475; Figure 2).

**Figure 2:**
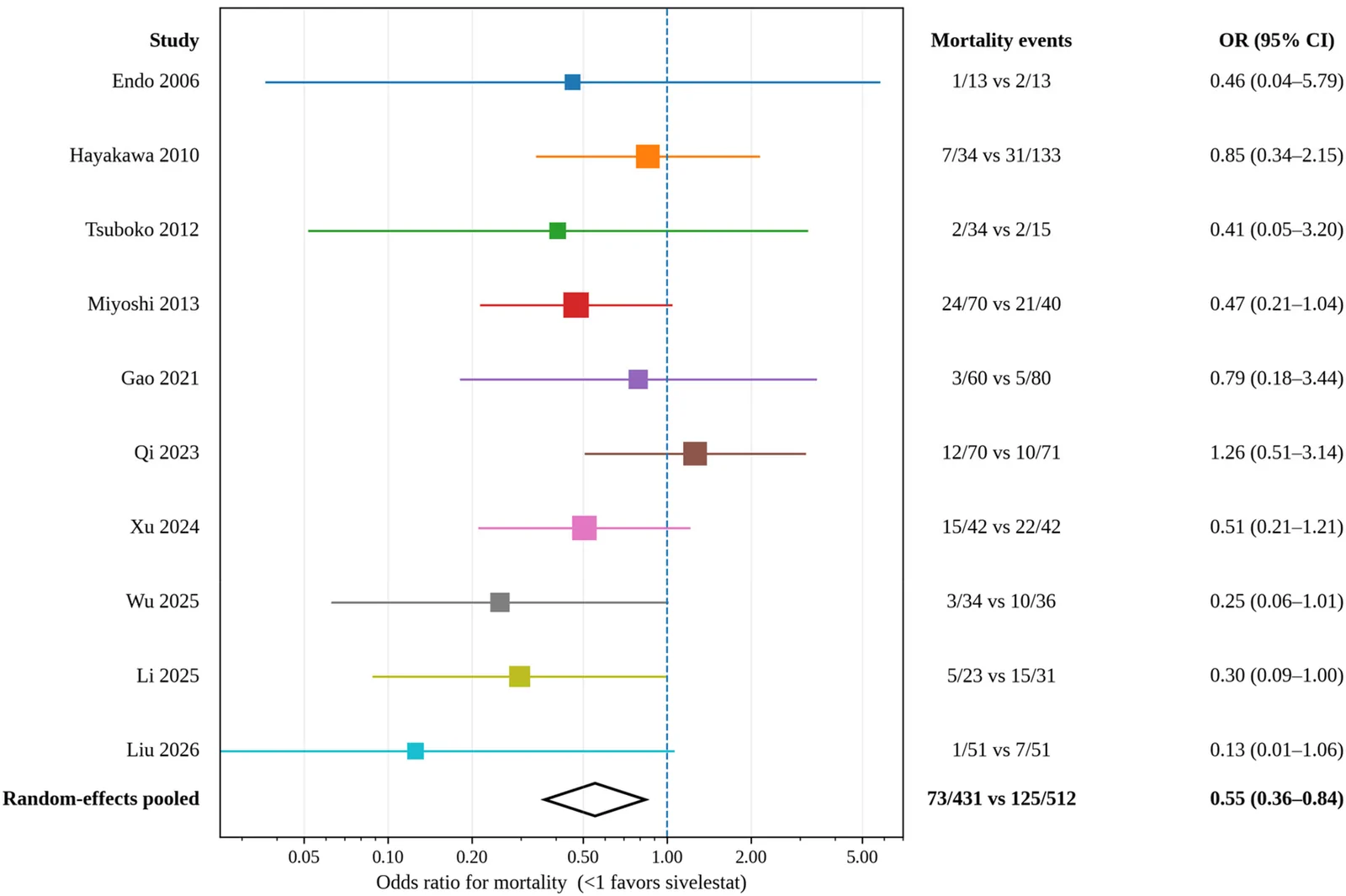
Random-effects meta-analysis of the effect of sivelestat on all-cause mortality in sepsis-induced ARDS. Square size approximately reflects random-effects weight; mortality event counts are shown on the right.

### 3.4 Subgroup analyses

After stratification by study design, the pooled OR was 0.37 for four RCTs (95% CI 0.16–0.86; *I^2^* = 0%) and 0.65 for six observational studies (95% CI 0.37–1.15; *I^2^* = 0%). The interaction test for study design yielded *P* = 0.169, indicating insufficient evidence that the true treatment effect differs between RCTs and observational studies.

For two studies with a study-level baseline *P/F* ratio <100 mmHg, the pooled OR was 0.42 (95% CI 0.21–0.86); for five studies with *P/F* 100–199 mmHg, the OR was 0.54 (95% CI 0.33–0.90); and for *P/F* 200–299 mmHg, only one study was available, with an OR of 1.26 (95% CI 0.51–3.14). A binary interaction test comparing <200 versus ≥200 mmHg yielded *P* = 0.080. Although the point estimates were directionally consistent with greater benefit at more severe hypoxemia, only one study was available in the ≥ 200-mmHg category; therefore, a true severity interaction cannot be confirmed.

Among three studies with control-group mortality >30%, the pooled OR was 0.44 (95% CI 0.26–0.75), compared with 0.63 (95% CI 0.35–1.14) among seven studies with control-group mortality ≤30%; the interaction test yielded *P* = 0.276. Point estimates were more favorable in higher-risk studies, but the current evidence does not provide statistically confirmatory evidence of effect modification.

For two studies in which both groups received ulinastatin as common background therapy, the pooled OR was 0.36 (95% CI 0.11–1.16), compared with 0.59 (95% CI 0.38–0.93) in the other eight studies; the interaction *P* value was 0.431. Current data are insufficient to show that ulinastatin significantly enhances or attenuates the mortality benefit associated with sivelestat. See Figure 3 for details.

**Figure 3:**
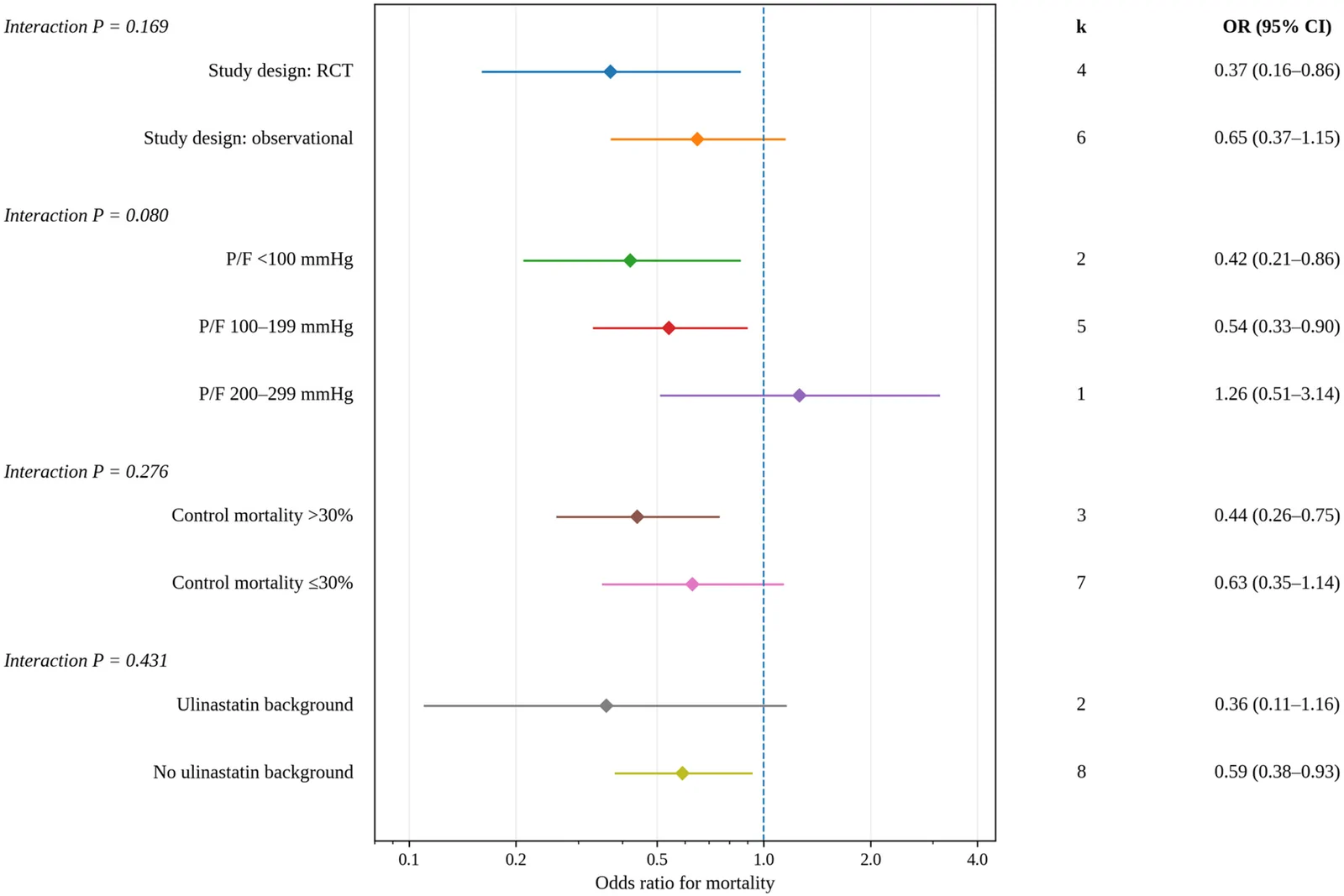
Exploratory subgroup analyses by study design, baseline oxygenation, baseline mortality risk, and ulinastatin background therapy.

### 3.5 Sensitivity analysis and publication bias

After omission of each study in turn, the pooled OR ranged from 0.48 to 0.59, and all Hartung-Knapp 95% CIs remained below 1, indicating that the overall mortality finding was not dependent on any single study (Figure 4). In the leave-one-out analysis, omission of *Li 2025* did not change the overall conclusion; the pooled OR remained within the range of 0.48–0.59, and all Hartung–Knapp 95% CIs remained below 1. However, because *Li 2025* protocolized methylprednisolone and APRV in both arms, this study informs the effect of sivelestat against a background of steroid and APRV therapy rather than against standard care alone.

**Figure 4:**
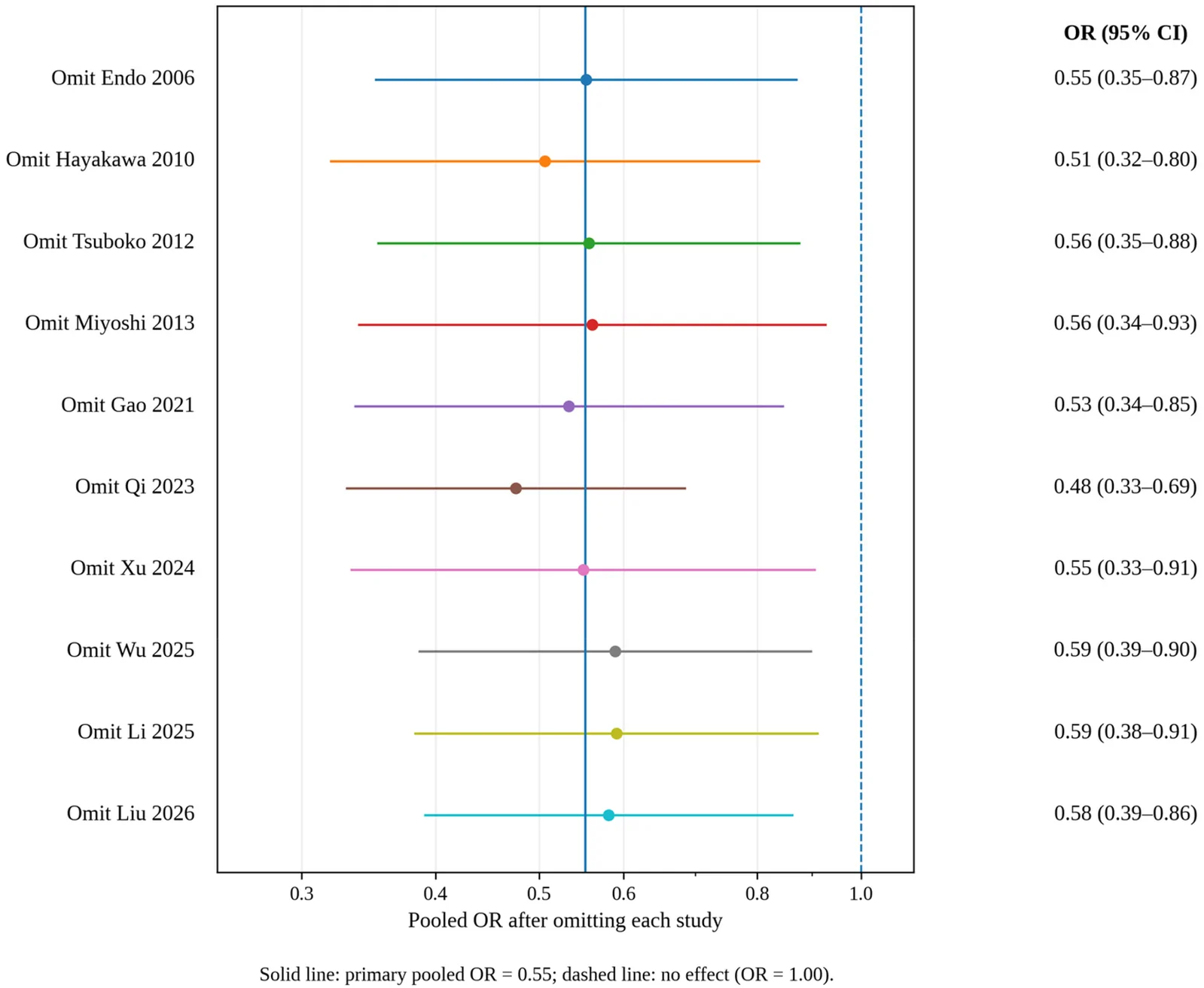
Leave-one-out sensitivity analysis. The solid line indicates the pooled OR from the primary analysis, and the dashed line indicates the line of no effect.

The funnel plot showed no marked extreme asymmetry; the intercept from Egger regression was −1.20 (*P* = 0.197; Figure 5). However, only 10 studies were available and eligibility was restricted to Chinese- and English-language publications; therefore, small-study effects and unpublished studies cannot be excluded.

**Figure 5:**
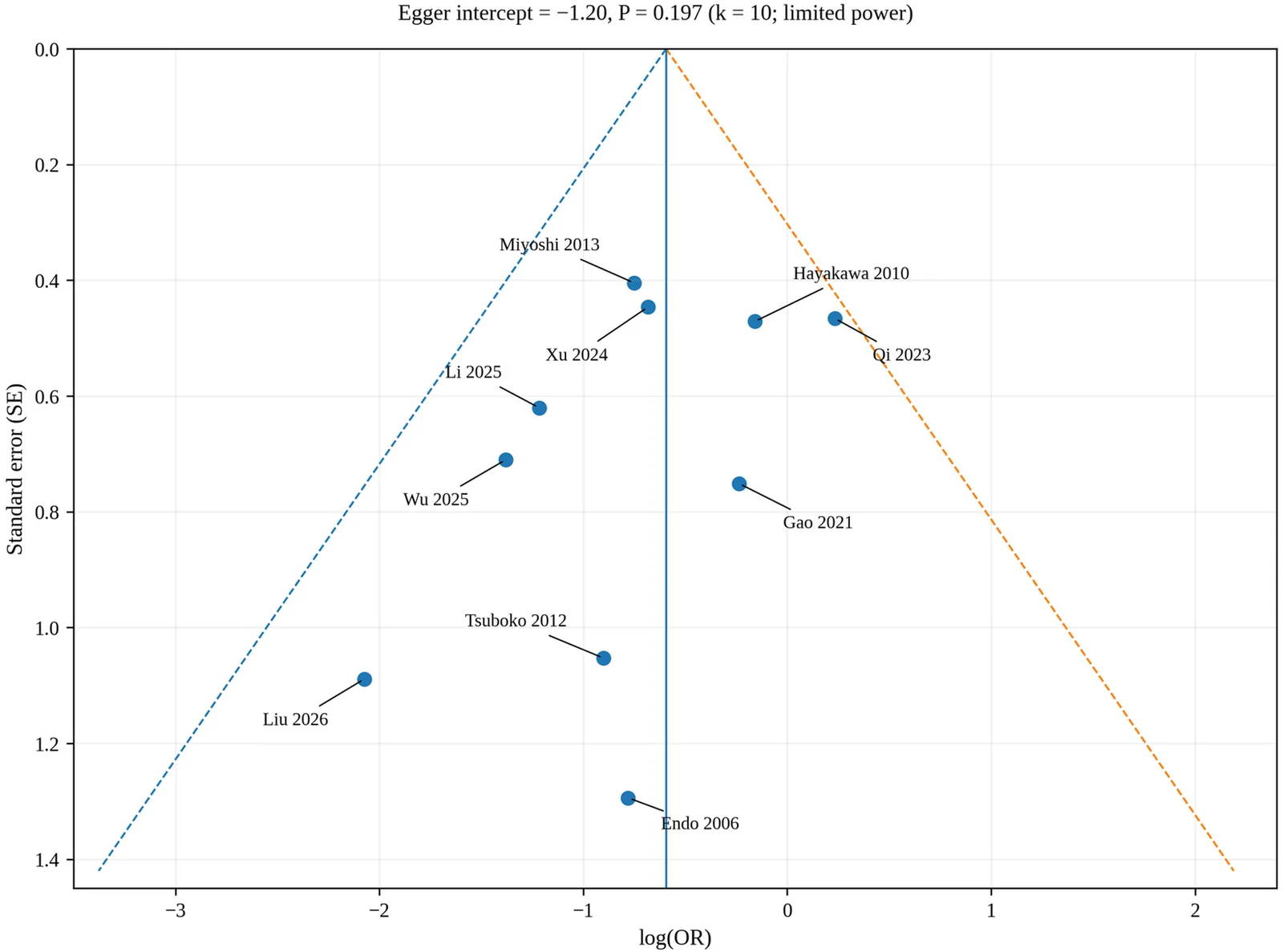
Funnel plot and egger test for the mortality outcome.

### 3.6 Secondary outcomes and evidence on inflammatory phenotypes

The previous meta-analysis by Zheng et al. showed that sivelestat may shorten ICU length of stay (SMD approximately −0.76) and duration of mechanical ventilation (SMD approximately −0.58), while improving the *P/F* ratio at multiple treatment time points (7). In the present update, the multicenter double-blind RCT by Wu 2025 reported a day-5 oxygenation response in 19/34 versus 7/36 patients and a 28-day survival hazard ratio favoring sivelestat. *Xu 2024* reported a duration of mechanical ventilation of 7.12 ± 0.59 versus 9.07 ± 1.09 days and an ICU stay of 9.19 ± 1.25 versus 11.36 ± 1.95 days, both favoring the sivelestat-containing combination regimen (22, 24).

Regarding inflammatory markers, Lv 2024, Li 2025, and Liu 2026 all suggested greater post-treatment reductions in IL-6, CRP, or other inflammatory indices (21, 25, 26). However, these studies mainly compared post-treatment levels or within-group changes and did not separately report treatment effects on mortality or clinical outcomes according to high- versus low-inflammatory phenotypes at baseline. Consequently, the current aggregate data cannot answer whether a high baseline IL-6 level predicts a greater response to sivelestat. This is a key evidence gap highlighted by the present review.

### 3.7 Safety

Previous systematic reviews did not identify a statistically significant increase in reported adverse events with sivelestat (7, 8). The updated studies likewise did not reveal a consistent signal of serious harm, but definitions of adverse events, monitoring periods, and completeness of reporting varied considerably. Accordingly, the most appropriate current conclusion is that no clear increase has been observed, rather than that complete safety has been established.

## 4 Discussion

### 4.1 Main findings

This updated meta-analysis applied a stricter etiologic definition than previous reviews and excluded COVID-19-only cohorts, non-septic ARDS, and comparisons in which the effect of sivelestat could not be isolated. Across 10 studies with exact mortality event counts and 943 patients, sivelestat was associated with an approximately 45% reduction in the odds of death, with very low statistical heterogeneity. Importantly, the RCT-restricted analysis retained a statistically significant benefit signal, indicating that the overall finding was not driven entirely by observational studies.

Compared with the overall mortality OR of approximately 0.62 reported by *Zheng 2025*, the point estimate in this strictly defined sepsis population was more favorable. However, this difference should not be interpreted as a true increase in treatment efficacy because the included studies, statistical models, and outcome time points differed. The 2026 RCT-restricted meta-analysis also supported a potential benefit of sivelestat in ALI/ARDS overall and suggested that sepsis-associated patients may represent a more appropriate enrichment population.

### 4.2 Do more severely ill patients benefit more?

The severity subgroups in the present study provide directional support for this hypothesis but not confirmatory evidence. The pooled point estimate was most favorable in studies with *P/F* < 100 mmHg, whereas only one study was available for *P/F* 200–299 mmHg and it did not show benefit; studies with control-group mortality >30% also showed a lower OR. However, neither the binary *P/F* interaction test nor the mortality-risk interaction test reached the conventional threshold for statistical significance, and these analyses used study-level mean values and are therefore subject to ecological bias. In other words, the proposition that more severely ill patients are most likely to benefit may serve as a stratification hypothesis for future trials, but it should not be presented as a clinical rule established by this meta-analysis.

### 4.3 Inflammatory phenotypes: a key knowledge gap

The mechanism of action of sivelestat provides strong biological plausibility for greater benefit in a high-NE or hyperinflammatory phenotype (4). Biological plausibility, however, is not equivalent to predictive evidence. Existing studies largely show reductions in IL-6, CRP, PCT, and related markers after treatment, but they do not report the mortality effect of sivelestat versus control stratified by baseline biomarker levels. Therefore, directly interpreting a reduction in IL-6 as evidence that patients with high IL-6 respond better would conflate predictive and pharmacodynamic evidence. The most informative future design would collect NE, IL-6/IL-8, CRP/PCT, and organ-function measures at baseline, prespecify high- and low-inflammatory subphenotypes, and formally test treatment-by-phenotype interactions.

International precision-medicine research in ARDS is increasingly promoting enrichment and platform trials based on reproducible phenotypes (27). Accordingly, the innovation of the present study is better framed not as proving efficacy in a known biomarker-defined subgroup, but as systematically identifying clinically testable enrichment factors and explicitly defining the current gap in predictive biomarker evidence.

### 4.4 Timing of treatment and combination therapy

Timing of treatment may also be important. A 2025 study compared different initiation times, but all patients received sivelestat; therefore, it cannot establish the causal effect of sivelestat relative to no sivelestat and can only support the hypothesis that earlier initiation merits further evaluation (28). Similarly, Yang 2024 compared sivelestat plus ulinastatin with sivelestat alone, which can be used to evaluate the incremental effect of ulinastatin but should not be included in the primary meta-analysis of sivelestat versus no sivelestat (29). In the present study, only *Xu 2024* and *Liu 2026*, in which the control treatment background was matched, were included in the ulinastatin-background subgroup.

In a non-septic population, a 2026 randomized cardiovascular-surgery trial also observed a signal that sivelestat reduced postoperative ARDS incidence together with reductions in IL-6 and NE (30). That study cannot be directly extrapolated to the treatment of sepsis-induced ARDS, but it mechanistically supports the feasibility of NE inhibition in hyperinflammatory lung injury.

### 4.5 Clinical implications

Given the current strength of evidence, sivelestat may be considered a potential adjunctive therapy for sepsis-induced ARDS rather than a replacement for standard ARDS and sepsis care, including lung-protective ventilation, prone positioning, fluid management, and source control. Optimal standard care for sepsis-induced ARDS, including lung-protective ventilation, prone positioning when indicated, fluid management, sedation, and neuromuscular blockade, should not be altered by sivelestat use; co-interventions should be delivered according to local protocols. If used clinically, priority may be given to patients most similar to those enrolled in existing studies: a clearly established septic etiology, early ARDS, more severe hypoxemia, and active inflammation, with concurrent assessment of hepatic and renal function, concomitant medications, and local availability. Current evidence is insufficient to establish hard thresholds such as restricting treatment only to patients with *P/F* < 100 or mortality risk >30%.

### 4.6 Limitations

First, the evidence base included both small RCTs and observational studies, with inconsistencies in randomization, blinding, baseline confounding, and treatment background. Although the RCT-restricted analysis retained the benefit signal, the total sample size remained limited. Second, mortality time points were not fully consistent across studies (14-day, 28-day, ICU, or in-hospital mortality). Third, reporting of P/F ratio, APACHE II, SOFA, and inflammatory markers was incomplete; subgroup analyses relied mainly on study-level means and cannot substitute for individual-patient-data (IPD) interaction analyses. Fourth, publication-bias testing was based on only 10 studies and therefore had limited power; language restriction may also have introduced selection bias. Fifth, safety outcomes were insufficiently reported and definitions were inconsistent, so rare or delayed adverse events cannot be excluded.Sixth, co-intervention data were sparsely reported. We could not determine whether the sivelestat and control groups received comparable prone positioning, sedation, or neuromuscular blockade, nor whether sivelestat was initiated before, during, or after proning. These variables may independently affect oxygenation and mortality and may therefore confound the observed treatment signal. Seventh, the evidence base was geographically concentrated in East Asia. All included studies were conducted in Japan or China, where sivelestat is approved and used in clinical practice. Standard care, ICU organization, availability of prone positioning, sedation and neuromuscular blockade practices, corticosteroid use, and ARDS case mix may differ from those in other regions. The observed mortality benefit may therefore not be directly generalizable to non-East Asian populations. Multinational randomized trials with prespecified sepsis-induced ARDS criteria are needed to confirm these findings. In addition, steroid/anti-inflammatory background therapy was inconsistent or unreported across studies. *Li 2025* protocolized methylprednisolone and APRV in both arms, whereas most other studies used standard care without explicit steroid reporting. Because these co-interventions were either balanced by design, unreported, or non-randomly distributed, residual confounding cannot be excluded, and the mortality estimate should be interpreted with this limitation in mind. Future trials should prespecify and report prone positioning, sedation, NMBA use, steroid exposure, and the timing of sivelestat initiation relative to these co-interventions.

## 5 Conclusions

Sivelestat was associated with a lower risk of all-cause mortality, and the RCT-restricted analysis continued to support this efficacy signal. Point estimates in studies with severe hypoxemia and high baseline mortality risk suggest potential value for clinical enrichment, but evidence from subgroup interaction tests remains insufficient. The most important current evidence gap is not the absence of additional data showing post-treatment reductions in IL-6, but the lack of treatment-effect estimates stratified by baseline phenotypes such as NE and IL-6, together with the lack of individual patient data. Nevertheless, co-intervention reporting was inadequate, and residual confounding by prone positioning, sedation, neuromuscular blockade, and steroid background remains possible. Future studies should use prespecified phenotypes, treatment-by-phenotype interaction testing, and standardized 28-day outcomes to determine which patients with sepsis-induced ARDS are most likely to benefit from sivelestat.

## Data Availability

The raw data supporting the conclusions of this article will be made available by the authors, without undue reservation.
